# Polymicrobial Late-Onset Knee Prosthetic Joint Infection Involving *Parvimonas micra*: A Case Report and Genomic Characterization

**DOI:** 10.3390/microorganisms14020413

**Published:** 2026-02-10

**Authors:** Mauro Jose Salles, Daniel Litardi Pereira, Ícaro Santos Oliveira, Fabiano Nunes Farias, Rafaela Carvalho Luz, Paola Cappellano, André Mário Doi, Raquel Bandeira da Silva

**Affiliations:** 1 Laboratório Especial de Microbiologia Clínica (LEMC), Division of Infectious Diseases, Department of Internal Medicine, Universidade Federal de São Paulo—UNIFESP, São Paulo CEP 04025-010, Brazil; 2 Musculoskeletal Infection Group, Department of Orthopedics and Traumatology, Escola Paulista de Medicina (EPM), Universidade Federal de São Paulo (UNIFESP), São Paulo CEP 04023-900, Brazil; 3 Disciplina de Infectologia, Faculdade de Ciências Médicas da Santa Casa de São Paulo, São Paulo CEP 01224-001, Brazil; 4 Real e Benemérita Associação Portuguesa de Beneficência SP, São Paulo CEP 01323-001, Brazil; 5 Microbiology Section, Grupo Fleury, São Paulo CEP 04344-070, Brazil; 6 Clinical Laboratory, Hospital Israelita Albert Einstein, São Paulo CEP 05652-900, Brazil; 7 Hospital Universitário Ciências Médicas (HUCM), Belo Horizonte CEP 30140-073, Brazil; 8 Directorate of Teaching and Research, Department of Internal Medicine, Faculdade Ciências Médicas de Minas Gerais (FCCMG), Belo Horizonte CEP 30120-016, Brazil

**Keywords:** anaerobic infection, prosthetic joint infection, *Parvimonas micra*, diagnosis, whole genome sequencing

## Abstract

We report a rare case of polymicrobial late-onset knee prosthetic joint infection (PJI) caused by *Parvimonas micra* and *Staphylococcus aureus*. An 80-year-old woman with multiple comorbidities presented, five years after total knee arthroplasty, with progressive pain and radiographic signs of prosthetic loosening. Synovial fluid analysis revealed marked neutrophilic inflammation, and intraoperative tissue cultures, including sonication fluid yielded both pathogens. Identification was confirmed by MALDI-TOF MS and whole-genome sequencing (WGS). The *P. micra* strain showed 97.2% identity to reference strain SAMN29629855, and carried virulence genes such as *groEL*, *tufA*, *clpP*, *ctrD*, *srtC4*, and *gaIE*, associated with oxidative stress response, adhesion, immune evasion, and biofilm formation. Resistance genes *vanW*, *vanT*, and *vanY* from the *van* operon were also detected, though *vanA* and *vanB* were absent. The patient underwent a two-stage revision surgery and a 12-week course of pathogen-targeted antimicrobial therapy, with complete resolution of symptoms and no recurrence after 12 months. This case highlights the overlooked pathogenicity of *P. micra* in chronic PJIs, especially in polymicrobial biofilm-related infections. The integration of WGS provided valuable insights into possible genetic characteristics of virulence determinants of this rare cause of PJI.

## 1. Introduction

Prosthetic joint infections (PJIs) remain the leading postsurgical complication, affecting up to 2% of primary and 4% of revision arthroplasties, profoundly impacting patients’ quality of life, increasing healthcare costs, and posing significant challenges to the management of implant-related infections [1,2,3].

The incorporation of advanced microbiological identification technologies has provided critical means to optimize clinical outcomes and resource utilization, thereby contributing to a reduction in adverse events in empirical therapy and mortality [4,5,6,7]. In this context, often overlooked or underrecognized anaerobic bacteria—such as *Cutibacterium acnes*, *Finegoldia magna*, *Fusobacterium* spp., *Prevotella* spp, and *Parvimonas micra*—are increasingly being recognized as true pathogens in PJIs [8]. Prosthetic joint infections associated with microorganisms of oral origin are uncommon and represent only a small subset of PJIs. A systematic review identified just 44 reported cases of PJIs temporally associated with dental procedures over several decades, with anaerobic Gram-positive bacteria from the oral microbiota accounting for approximately 18% of isolated pathogens, highlighting both the rarity and the likely underrecognition of these organisms [9]. We report on the case of an elderly patient who developed a late-onset total knee arthroplasty (TKA) coinfection involving *Staphylococcus aureus* and *P. micra*. Comprehensive whole-genome sequencing (WGS) and phenotypic characterization of *P. micra* were performed to elucidate its clonality and pathogenic potential, including adhesion, intracellular invasion, biofilm formation, and antimicrobial resistance mechanisms within the framework of PJI. This study was reviewed and approved by the Research Ethics Committee (CEP) of Instituto Fleury/SP, Brazil, through Plataforma Brasil, and received ethical approval under Opinion Number 7.017.378 (CAAE: 77143523.0.0000.5474), confirming compliance with all applicable ethical standards for research involving human data.

## 2. Case Report

**Figure 1:**
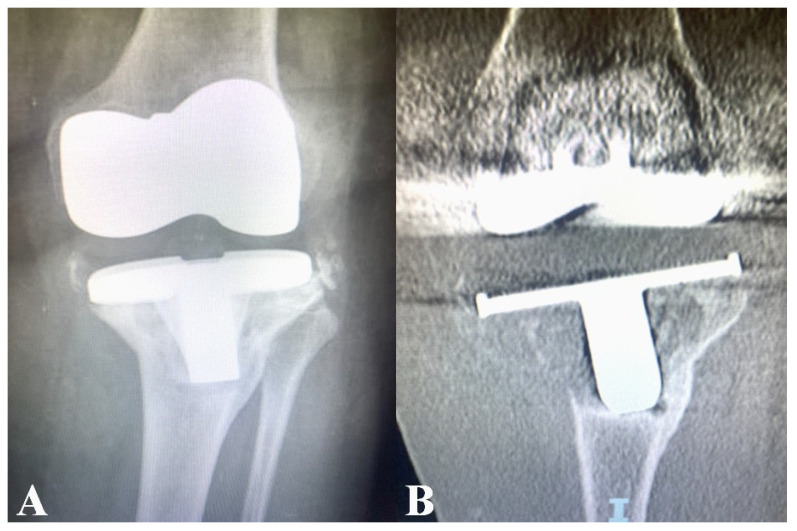
(**A**,**B**) Patient’s X-ray and CT scan of left knee showing implant loosening.

An 80-year-old female with a complex medical history—including active smoking, diabetes mellitus, atrial fibrillation, morbid obesity (BMI > 40), and a history of successfully treated breast cancer—presented with left knee osteoarthritis. She underwent TKA while exhibiting ongoing gingivitis, which was not considered clinically significant at the time. Three months after TKA, she underwent gastrectomy for the treatment of a perforated ulcer. Five years later, the patient presented with insidious left knee pain that had progressively become disabling, accompanied by radiographic findings suggestive of prosthetic loosening (Figure 1A,B).

**Figure 2:**
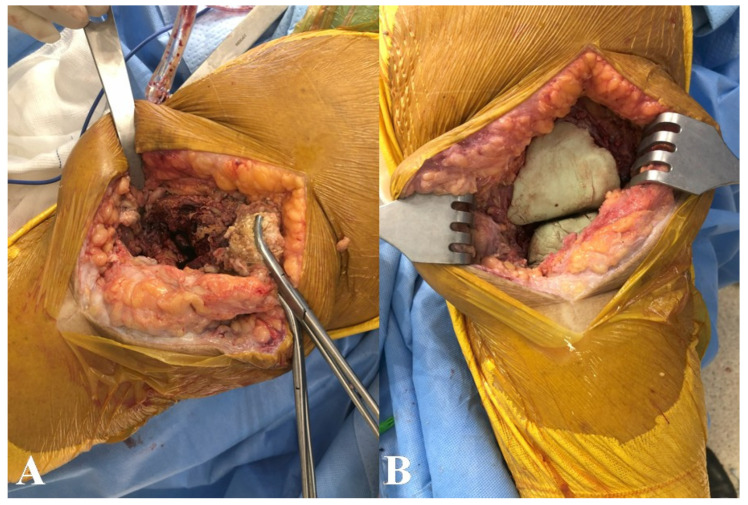
(**A**,**B**): Surgical debridement, tissue sample collection for cultures, and PMMA spacer loaded with antibiotics implanted.

Laboratory workup revealed a white blood cell count of 10,420/µL and a C-reactive protein level of 6.9 mg/L. Synovial fluid analysis demonstrated 51,195 leukocytes/µL, with 83% neutrophils. A two-stage revision surgery was performed. A polymethylmethacrylate (PMMA) spacer loaded with vancomycin and gentamicin was implanted (Figure 2A,B), and empirical intravenous ceftriaxone was initiated.

**Figure 3:**
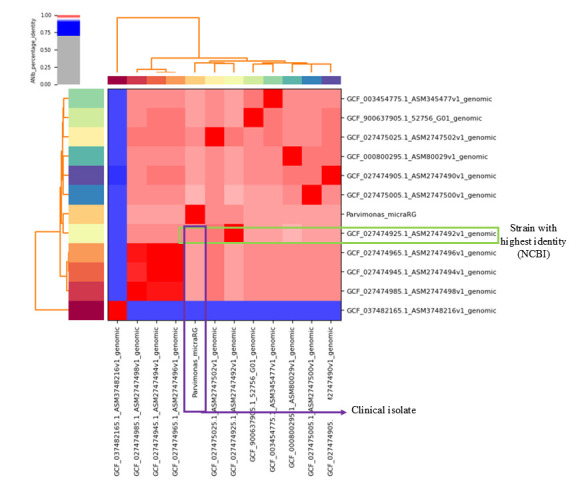
Genome sequencing of *P. micra*: The dendrogram includes several isolates deposited in the NCBI database, identified by their accession numbers (GCF). The clinical isolate is labeled as Parvimonas_micraRG (purple box). The isolate showing the highest genetic identity with the clinical strain is highlighted by the green box. The scale at the top of the dendrogram represents the percentage of genetic identity among the analyzed isolates, with increasing red color intensity indicating higher levels of genetic similarity.

Intraoperative cultures of periprosthetic tissues and synovial and sonication fluids were processed using anaerobic culture in both solid and liquid media, yielding *P. micra* and *S. aureus*, both confirmed by matrix-assisted laser desorption/ionization time-of-flight mass spectrometry (MALDI-TOF MS) and WGS (Figure 3). Synovial fluid cytology with white blood cell counts higher than 3000 cells/µL and a percentage of polymorphonuclear cells (PMN) higher than 80%, with the same pathogen yield in deep tissue and sonication fluid of the retrieved implant, supported that it represents a true pathogen rather than contamination, which confirms the diagnosis of PJI according to the European Bone and Joint Infection Society (EBJIS)’s PJI definition [10]. In addition, the *P. micra* susceptibility profile was confirmed by antimicrobial susceptibility testing (AST) using E-test and/or microdilution, providing MIC values (mg/L). The isolate demonstrated a fully susceptible anaerobic phenotype, with very low MICs to penicillin G ≤ 0.016 mg/L (S), metronidazole 0.047 mg/L (S), and clindamycin 0.19 mg/L (S). Antimicrobial susceptibility testing for *Staphylococcus aureus* was performed using a standard automated susceptibility testing system, with interpretation according to EUCAST criteria. The isolate was susceptible to all tested agents except penicillin, confirming methicillin susceptibility

Whole-genome sequencing was carried out with paired-end Illumina NextSeq550 platform with NextTera kit (Illumina, Inc., San Diego, CA, USA) producing 150 bp read fragments. Raw sequence data was submitted to metagenomic analysis software, Varsmetagen^®^ (v2.3, cloud-based pipeline), that carried a WGS pipeline for classification and generation of complete bacterial genomes. Briefly, a total of 3 million reads were filtered and classified with Kraken2 (Version v2.17.0) using a custom database. Reads were assembled with SPAdes v4.1 followed by a second round of classification with kraken2.

MetaWRAP (Version v1.3.2) was employed to group contigs into bins, which were subsequently classified as metagenome-assembled genomes (MAGs) using GTDB-Tk (GTDB R214). Over 1 million reads and 17 assembled sequences (contigs) were classified as *Parvimonas micra*. The final MAG had a total of 1.6 Mb, with a 248× depth coverage, and is available in GenBank (SAMN54754354).

**Figure 4:**
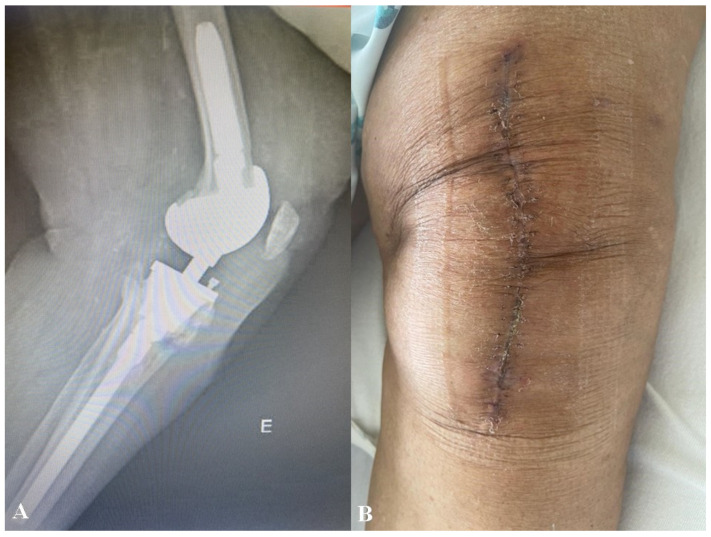
Revision left knee arthroplasty with no clinical or radiological signs of infection. (**A**) Radiological imaging demonstrating a left knee revision prosthesis with no evidence of loosening; (**B**) surgical wound from the left knee revision arthroplasty showed no signs of infection.

After 12 weeks of targeted antimicrobial therapy, the PMMA spacer was removed, ceftriaxone was discontinued, and a revision prosthesis was successfully implanted. During 12 months of follow-up, the patient exhibited restored motor function and no evidence of infection recurrence (Figure 4).

## 3. Literature Review and Discussion

We describe the first polymicrobial chronic knee PJI caused by the association of biofilm-producing *P. micra* and *S. aureus*. Formerly known as *Micromonas micros* and *Peptostreptococcus micros*, *P. micra* is a slow-growing, fastidious, strictly anaerobic, Gram-positive coccus that colonizes the human gastrointestinal tract and is typically associated with periodontitis [11]. However, under certain conditions, it can transition into an invasive phenotype, leading to bacteremia associated with a variety of infections—including meningitis, central nervous system abscesses, panophthalmitis, endocarditis, pulmonary empyema, spondylodiscitis, osteomyelitis, pyomyositis, and native joint arthritis—and has also been implicated in colorectal oncogenesis [12,13,14,15,16,17,18,19,20,21,22,23].

The paucity of symptoms observed in the present case aligns with reports in the literature. A systematic review of *P. micra* infection routes found fever in fewer than 50% of reported cases, with nonspecific constitutional symptoms such as general malaise and anorexia being frequently noted. Pain was the most reported symptom, primarily associated with musculoskeletal inflammation [11]. Notably, only five cases of *Parvimonas micra*-related PJI following TKA have been reported in the literature to date [24,25,26,27,28]. To further illustrate the association between *P. micra* infection and arthroplasty, a comparative table summarizing clinical findings, diagnostic methods, and treatment strategies from reported cases is provided [24,25,26,27,28,29,30,31,32,33,34,35] (Table 1).

We speculate that distinct sources of *P. micra* and *S. aureus* contributed to the development of PJI, with chronic periodontitis or gut translocation serving as potential origins for the former, and nasal colonization for the latter [36]. Indeed, *P. micra* often exhibits mutualistic relationships with other bacteria within biofilms, including *Porphyromonas gingivalis*, *Fusobacterium nucleatum*, *Actinomyces naeslundii*, members of the order Enterobacterales, and various *Streptococcus* species [37,38,39,40]. These microbial communities, supported by an extracellular matrix primarily composed of exopolysaccharides and lipoteichoic acids, contribute to microbiome stability and pathogenic potential. As demonstrated by Liu et al., regulation of the *dlt*A gene in *P. micra* modulates lipoteichoic acid expression within the biofilm matrix, directly influencing biofilm formation and bacterial growth [41].

Beyond the descriptive comparison of previously reported cases, the data summarized in Table 1 allow a deeper interpretation of the clinical behavior and pathogenic relevance of *Parvimonas micra* in PJI. The remarkably small number of documented cases, despite the widespread use of joint arthroplasty and the well-recognized prevalence of anaerobic bacteria within the human microbiota, strongly suggests that *P. micra*–associated PJIs are substantially underdiagnosed rather than genuinely rare. This discrepancy likely reflects a longstanding diagnostic bias towards aerobic and rapidly growing organisms, together with the limited routine use of prolonged anaerobic incubation, implant sonication, and molecular diagnostic techniques in many orthopedic centers [8].

The temporal evolution of reported cases further supports this interpretation. Earlier publications relied almost exclusively on conventional culture-based methods, often resulting in delayed or incomplete pathogen identification. In contrast, more recent studies increasingly incorporate advanced technologies such as MALDI-TOF MS, metagenomic sequencing, and whole-genome sequencing [27,31]. This shift mirrors broader developments in clinical microbiology and indicates that improved diagnostic resolution has played a pivotal role in recognizing *P. micra* as a true pathogen rather than an incidental or contaminant organism. Its repeated recovery from deep periprosthetic tissues [29,32], synovial fluid [25,26,27,28], and sonication fluid [30] across independent cases further substantiates its pathogenic significance.

Clinically, a consistent pattern of late-onset infection with indolent presentation emerges. Unlike acute PJIs caused by highly virulent organisms, *P. micra* infections typically manifest as isolated or progressive joint pain, functional decline, and radiological evidence of prosthetic loosening, frequently in the absence of systemic inflammatory features [31,35]. Such presentations increase the risk of misdiagnosis as aseptic failure and may delay appropriate microbiological investigation and definitive management. These observations underscore the importance of maintaining a high index of suspicion for anaerobic pathogens in cases of chronic prosthetic dysfunction, particularly in elderly patients and those with substantial comorbidities that may predispose them to haematogenous seeding or impaired immune responses.

From a therapeutic perspective, published cases indicate that *P. micra* remains largely susceptible to commonly used anti-anaerobic agents, including β-lactams, clindamycin, and metronidazole. The absence of clinically relevant resistance is consistent with existing microbiological data and supports the effectiveness of targeted antimicrobial therapy once the organism is accurately identified. Nevertheless, the frequent requirement for revision surgery, especially two-stage exchange procedures in late-onset infections, highlights the central role of biofilm formation and implant-associated persistence in disease pathogenesis. These findings reinforce the principle that surgical decision-making should be driven primarily by infection chronicity, implant stability, and host-related factors rather than antimicrobial susceptibility alone [1].

An additional challenge arises from the polymicrobial potential of *P. micra*, which may further complicate both diagnosis and management. Anaerobic cocci are known to engage in synergistic interactions within polymicrobial biofilms, enhancing microbial survival, immune evasion, and tissue destruction. In this context, failure to detect *P. micra* may result in incomplete pathogen coverage and suboptimal antimicrobial strategies. The selective integration of molecular diagnostics into routine workflows may therefore not only improve detection rates but also strengthen antimicrobial stewardship by enabling more precise, pathogen-directed therapy [8].

Regarding clinical outcome, most reported patients achieved favorable results following appropriate surgical and antimicrobial intervention. Infection resolution was commonly attained through adequate surgical source control combined with prolonged, targeted antimicrobial therapy. Recurrence was rarely reported, and adverse outcomes, when present, were predominantly related to host factors such as advanced age or cardiovascular comorbidities rather than microbiological failure [26]. These observations suggest that delayed recognition, rather than intrinsic pathogen virulence, represents the principal determinant of unfavorable outcome in *P. micra*-associated PJIs.

Despite these insights, important knowledge gaps remain. Systematic data on the true incidence of *P. micra* in PJIs, its virulence determinants in vivo, and its interactions with other microorganisms within prosthetic biofilms are still limited. Furthermore, the predominance of single case reports and small case series constrains the ability to draw robust conclusions regarding optimal surgical or antimicrobial strategies. Future multicenter studies integrating detailed clinical data with advanced microbiological and genomic analyses are therefore essential to better define the epidemiological burden and clinical significance of this organism in implant-associated infections.

Preventive considerations for PJI caused by oral pathobionts have been advocated for, and they should be guided by current evidence rather than routine dental interventions. Although *P. micra*, another Gram-positive anaerobic cocci commonly colonizing the oral cavity, has been implicated in hematogenous seeding of joints in selected cases, particularly in the presence of poor oral hygiene, gingivitis, or periodontitis, PJIs attributable to oral anaerobes remain rare when compared with those caused by staphylococci [28]. A recent international consensus and meta-analysis published in 2025 found no statistically significant reduction in surgical site infection or PJI rates among patients undergoing major orthopedic procedures who received routine preoperative dental screening compared with those who did not [42]. Consistently, current clinical practice guidelines from the American Academy of Orthopaedic Surgeons and the Infectious Diseases Society of America do not recommend routine dental clearance or antibiotic prophylaxis prior to dental procedures in patients with hip or knee arthroplasties, even in high-risk populations, due to the low incidence of oral flora-related PJI and the lack of demonstrated benefit [43]. Preventive strategies should therefore focus on established perioperative measures, including optimization of comorbidities, standard perioperative antimicrobial prophylaxis, and skin and nasal decolonization [44,45]. Perhaps, in selected patients such as elderly individuals and those with poor dentition, active periodontal disease, prior PJI, or significant immunosuppression, an individualized risk assessment and multidisciplinary approach may be reasonable, while maintaining good oral hygiene and prompt treatment of dental infections remains universally recommended [42]. Despite continuous technical advancements, conventional culture-based identification methods often result in low microbial yields and delayed diagnoses [28,41]. In addition, due to the polymicrobial contexts in which it is often found, *P. micra* remains poorly characterized in implant-associated infections. In a literature review, Watanabe et al. found 126 cases of bacteremia caused by Gram-positive anaerobes between 2016 and 2018, a substantial increase compared to the 70 cases reported between 2013 and 2015, largely attributed to the introduction of techniques such as MALDI-TOF MS and 16S rRNA gene sequencing [46].

Genomic analyses of *P. micra* have revealed potential virulent genes that may contribute to its pathogenicity [47,48]. The BLAST search (https://blast.ncbi.nlm.nih.gov/Blast.cgi, accessed on 26 April 2025) revealed a 97.2% identity with a strain isolated from a Spanish biorepository, SAMN29629855, representing the genome with the highest similarity. The constructed dendrogram (Figure 4) highlighted virulent genes such as *gro*EL, *tuf*A, *clp*P, *ctr*D, *srt*C4, and *gaI*E, which are associated with adhesion, survival under oxidative stress, and immune modulation. Pinheiro et al., in a metatranscriptomic analysis of the endodontic microbiome, showed that the *tuf*A was among the most highly expressed genes in the context of periodontitis pathogenesis, primarily contributing to bacterial adhesion, promoting coaggregation/coadhesion with other bacterial species and epithelial cells of the oral mucosa, thereby facilitating tissue invasion and dissemination [49]. The activation of cellular proliferation signaling pathways by *P. micra* has even been implicated in colorectal carcinogenesis, as demonstrated by Chang et al. in an animal model [17]. Additionally, *gro*EL chaperonins play a key role in biofilm formation by enabling bacterial survival under neutrophil-generated oxidative stress [47,49]. Proteases such as gingipains, produced during polymicrobial interactions, further contribute to virulence by cleaving proinflammatory cytokines—including IL-2, IL-1β, TNF-α, IL-6, and IL-8—thereby creating irregularities in the host’s immune response, favoring local dysbiosis and tissue destruction. Indeed, intense bone tissue reabsorption was clearly identified surrounding the knee prosthesis of our clinical case. Other pathogenic mechanisms include the remodeling of the biofilm in favor of inflammatory bacteria, evasion of innate immunity, and proteolytic degradation of immunoglobulins (IgG, IgA, and IgM) and complement proteins C3 and C4 [48,49].

In the genome analysis, resistance genes from the van operon (*van*W, *van*T, and *van*Y) were detected. However, none of these genes are directly associated with glycopeptide resistance, particularly in the absence of key determinants such as *vanA* or *vanB*, which are the main drivers of high-level vancomycin resistance through modification of the antimicrobial binding target. Pinheiro et al. found an association of *van*A and *van*B genes with resistance to glycopeptides by altering the antimicrobial binding site, but these genes were lacking in the clinical strain analyzed [49]. Interpretation of sequencing results should always be based on clinical context, as the method can identify DNA from colonizing organisms, which is one of the limitations of the WGS technique. Consistently, MIC-based phenotypic susceptibility testing did not demonstrate glycopeptide resistance in the isolate. Therefore, the *van* operon finding in this case was interpreted as a genomic observation with potential epidemiological relevance, rather than evidence of expressed resistance requiring modification of therapy. Importantly, sequencing data should always be interpreted within the clinical context, as WGS may identify resistance-associated genetic elements that are not expressed or that lack functional impacts, and may also capture DNA from colonizing organisms. In the present case, concordant identification of *P. micra* by both culture and WGS, together with the clinical scenario, supported its role as a true pathogen.

This report is limited by its single-case design, which inherently restricts the generalisability of the clinical, microbiological, and genomic observations presented. Although whole-genome sequencing identified several genes previously associated with virulence and antimicrobial resistance in *Parvimonas micra*, these findings are based on genomic annotation and homology and were not supported by functional validation. No experimental assays, such as biofilm formation studies or gene expression analyses, were performed; therefore, the presence of these genes should not be interpreted as definitive evidence of their expression or biological activity in vivo. Our case report is unique in that it demonstrates an unusual association of *P. micra* and *S. aureus* with a chronic onset of PJI. The clinical presentation was atypical for a polymicrobial Gram-positive PJI, as progressive local pain was the unique presenting symptom. It is known that anaerobic bacteria account for 15% of the etiological agents in PJIs, as reported by Zeller et al. in a retrospective cohort study, although this figure is likely an underestimation [50]. Cases of PJIs caused by anaerobic species such as *Cutibacterium, Clostridium, Veillonella, Bacteroides, Finegoldia, Fusobacterium*, and *Actinomyces* have been documented, occurring either as monomicrobial or polymicrobial infections. These can be found in either monomicrobial or polymicrobial infections. Therefore, the advent of next-generation sequencing methods has greatly optimized early diagnosis and guided therapy for affected patients, as illustrated by the present case, and has enhanced understanding of the phenotypic expression of virulent genes in the context of implant-associated infections [51,52].

## 4. Conclusions

This case report highlights the underestimated pathogenic role of *Parvimonas micra* in chronic PJI, particularly in late-onset and polymicrobial biofilm-associated scenarios. The association of *P. micra* with Staphylococcus aureus in a delayed knee prosthetic joint infection likely underscores the capacity of this anaerobic organism, traditionally regarded as a commensal of the oral and gastrointestinal microbiota, to behave as a clinically relevant pathogen in implant-related infections. The indolent clinical presentation observed, characterized mainly by progressive pain and radiographic evidence of prosthetic loosening in the absence of prominent systemic inflammatory signs, illustrates the diagnostic challenges inherent to anaerobic PJIs. The consistent recovery of *P. micra* and S. aureus from synovial fluid, multiple deep intraoperative tissue samples, and sonication fluid, together with marked neutrophilic inflammation, supports their role as true pathogens and reinforces the importance of optimized microbiological diagnostic strategies, including prolonged anaerobic culture and advanced identification techniques. The integration of MALDI-TOF MS and whole-genome sequencing was pivotal for accurate pathogen identification and characterization. Genomic analysis of the isolated *P. micra* strain revealed the presence of likely genomic traits associated with infection, including genes related to adhesion, oxidative stress response, immune modulation, and biofilm formation, which may contribute to persistence in the prosthetic joint environment. Whole-genome sequencing also enabled high-resolution comparison with reference strains and identification of resistance-associated elements of the van operon, although the absence of phenotypic glycopeptide resistance emphasizes the need to interpret genomic findings within the clinical and microbiological context. The favorable clinical outcome achieved through a two-stage revision procedure combined with prolonged, pathogen-targeted antimicrobial therapy highlights the effectiveness of a multidisciplinary approach integrating orthopedic surgery, infectious diseases, and clinical microbiology. Overall, this case reinforces that *Parvimonas micra* should not be overlooked in chronic prosthetic joint infections and demonstrates that the incorporation of whole-genome sequencing may enhance biological understanding, refine diagnostic accuracy, and support precision diagnostics in rare causes of prosthetic joint infection.

## Figures and Tables

**Table 1:** Summary of 12 cases of *P. micra* causing PJI in the current literature.

Reference	Affected Joint	Clinical Presentation	Diagnosis Methods	Antimicrobial Treatment	Surgical Management	Clinical Outcome
Randall et al. 2020 [24]	Knee	Progressive stiffness	Anaerobic culture of synovial fluid; isolation after prolonged incubation	Intravenous (IV) ceftriaxone for 6 weeks	Two-stage exchange arthroplasty over a 3-month period	Additional surgery required 3 months later due to instability
Stoll et al. 1996 [25]	Knee	Low-grade fever with a painful, warm, and swollen knee	Culture of aspirated knee exudate	Intravenous imipenem for 6 weeks, followed by oral (PO) clindamycin plus rifampicin for 2 weeks	Repeated needle aspiration and joint irrigation	Favorable
Bangert et al. 2019 [26]	Knee	Pain, local warmth, and moderate joint effusion	Culture of aspirated knee exudate	Moxifloxacin administered prior to surgery	Prosthetic replacement	Postoperative death due to a coronary event
Cao et al. 2020 [27]	Knee	Severe progressive swelling and pain	Anaerobic culture of synovial fluid using blood culture bottles and anaerobic agar, with identification by MALDI-TOF MS	Not reported	Debridement, antibiotics, and implant retention (DAIR)	Favorable
Piñeiro et al. 2021 [28]	Knee	Pain and knee swelling	Anaerobic culture of synovial fluid with isolation after prolonged incubation	Intravenous ceftazidime plus vancomycin, followed by oral amoxicillin–clavulanate for 8 weeks	Two-stage exchange revision with cemented implant	Favorable
Bartz et al. 2005 [29]	Hip	Pain	Aerobic and anaerobic culture of surgical tissue samples	Oral clindamycin 300 mg four times daily	Prosthesis removal and one-stage exchange surgery	Favorable
Rieber et al. 2020 [30]	Hip	Pain six months after prosthetic joint surgery	Joint tissue cultures and sonication fluid analysis	Not reported	Not reported	Not reported
Huang et al. 2019 [31]	Hip	Mild and intermittent local pain	Metagenomic next-generation sequencing (mNGS) performed on synovial fluid	Intravenous piperacillin–tazobactam (Pipe/tazo) for 2 weeks, followed by oral amoxicillin–clavulanate for 8 weeks	Two-stage exchange surgery with a cemented implant	Favorable
Petrini et al. 1979 [32]	Hip	Pain, fever, prosthetic loosening with local fistula	Anaerobic cultures obtained preoperatively from the depth of the fistula, with periprosthetic tissue and pus cultures collected at reoperation	Not reported	Not reported	Not reported
Bohra et al. 2018 [33]	Hip	Pain eight months after prosthetic joint surgery	Joint cultures	Intravenous vancomycin for 2 weeks	Revision surgery	Favorable
Marmor et al. 2020 [34]	Hip	Fistulised chronic prosthetic hip infection	Preoperative joint aspiration	Not reported	One-stage exchange revision arthroplasty	Favorable
Aggarwal et al. 2021 [35]	Hip	Pain five years after prosthetic joint implantation	Culture of aspirated exudate and intraoperative joint fluid cultures	Intravenous ceftriaxone for 6 weeks, followed by oral penicillin V for 6 weeks	Debridement, antibiotics, and implant retention (DAIR)	Favorable

IV: Intravenous; PO: oral; Pipe/tazo: Piperacillin/tazobactam; DAIR: Debridement, antibiotic, and implant retention.

## Data Availability

The data presented in this study are available on request from the corresponding author. The data are not publicly available due to it is a description of the patient data from the medical record.
